# Strongyloides Hyperinfection in a Renal Transplant Patient: Always Be on the Lookout

**DOI:** 10.1155/2017/2953805

**Published:** 2017-02-20

**Authors:** Murtaza Mazhar, Ijlal Akbar Ali, Nelson Iván Agudelo Higuita

**Affiliations:** ^1^Department of Internal Medicine, Suite 6300, 800 Stanton L Young Boulevard, Oklahoma University Health Sciences Center, Oklahoma City, OK 73104, USA; ^2^Department of Infectious Diseases, Suite 7300, 800 Stanton L Young Boulevard, Oklahoma University Health Sciences Center, Oklahoma City, OK 73104, USA

## Abstract

We present a case of a 71-year-old Vietnamese man with chronic kidney disease secondary to adult polycystic kidney disease. He had been a prisoner of war before undergoing a successful cadaveric renal transplant in the United States. He presented to clinic one year after the transplant with gross hematuria, productive cough, intermittent chills, and weight loss. Long standing peripheral eosinophilia of 600–1200/*μ*L triggered further evaluation. A wet mount of stool revealed* Strongyloides stercoralis *larvae. A computed tomography (CT) of chest showed findings suggestive of extension of the infection to the lungs. The patient was treated with a three-week course of ivermectin with complete resolution of signs, symptoms, peripheral eosinophilia, and the positive IgG serology. Strongyloides infection in renal transplant patient is very rare and often presents with hyperinfection, associated with high mortality rates. The American Transplant Society recommends pretransplant screening with stool examination and* Strongyloides stercoralis* antibody in recipients and donors from endemic areas or with eosinophilia. It is imperative that healthcare professionals involved in the care of these individuals be cognizant of these recommendations as it is a very preventable and treatable entity.

## 1. Introduction

*Strongyloides stercoralis* is an intestinal nematode that is unique in its ability to complete its entire life cycle in the human host. The infection can be therefore present for decades without causing symptoms. In immunocompromised hosts, progression of the chronic intestinal infection can lead to a potentially fatal hyperinfection syndrome. We describe an interesting case of an immunocompromised Vietnamese man with likely hyperinfection two decades after initial exposure to the parasite.

## 2. Case Presentation

A 71-year-old Vietnamese man was diagnosed with chronic kidney disease secondary to adult polycystic kidney disease two years after immigrating to the United States. He had been a prisoner of war for 14 years in the jungles of Vietnam 5 years before arriving to the US. He underwent a successful cadaveric renal transplant 19 years after immigration from a US-born donor. No other details from the donor were available. The patient's immunosuppressive regimen consisted of prednisone, mycophenolate mofetil, and tacrolimus. One year after the transplant, he presented to his nephrologist for the evaluation of gross hematuria. He also reported cough productive of yellow sputum, shortness of breath, intermittent chills, weight loss, and fatigue of several weeks duration. The physical exam was notable for the presence of rales on both lung bases, left more than right. The abdomen was soft with no hepatosplenomegaly or masses. No rash was noted.

**Figure 1:**
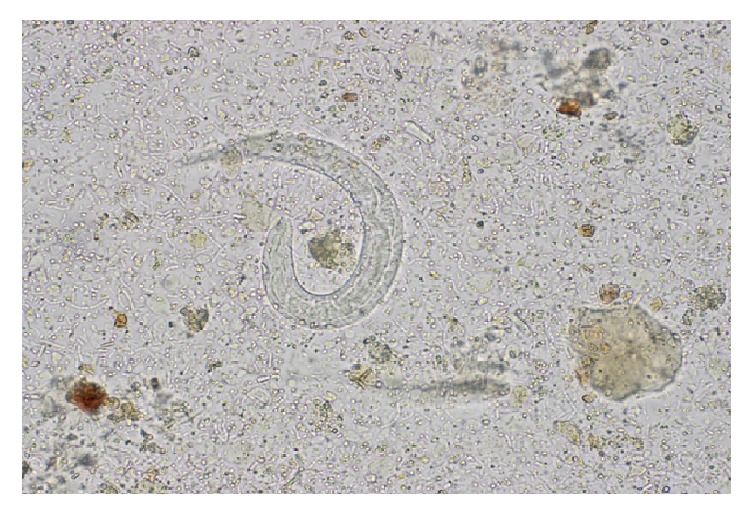
Wet mount of stool showing Strongyloides larvae.

A urine analysis showed hematuria, pyuria, and proteinuria. The urine culture failed to isolate an organism. A cystoscopy showed no abnormalities and a cytology of a bladder wash showed atypical inflammatory cells. He was empirically treated with a ten-day course of cephalexin for a presumed urinary tract infection with resolution of the hematuria. Peripheral eosinophilia of 1200/*μ*L (normal range 0–500/*μ*L) [1] was noted on the complete blood count (CBC) and further evaluation of the records showed that he had had intermittent mild peripheral blood eosinophilia (600–800/*μ*L range) for at least 7 years before transplantation. No screening for* S. stercoralis* had been done and screening for HTLV-1 was negative. A* Strongyloides stercoralis* IgG was therefore obtained which yielded a positive result of 1.96 IU (<1 IU is considered negative). Numerous Strongyloides larvae were easily identified on a wet mount of a stool specimen by their morphological characteristics of a large genital primordium and short buccal capsule (Figure 1). Larvae were not detected in the urine or sputum despite repeated testing. Blood cultures were negative. A computed tomography (CT) of the chest showed mild peribronchial thickening with a mosaic pattern within the bilateral lobes and ill-defined patchy nodular opacity within the posterior segment of the right upper lobe suggestive of infectious etiology. The patient was treated with a three-week course of ivermectin at a dose of 200 *μ*g/kg/day with resolution of his symptoms before the end of therapy and therefore a bronchoalveolar lavage was not pursued. Larvae were no longer visualized in 3 consecutive stool specimens using an agar culture method at the end of treatment and 2 weeks after completion of therapy. The peripheral eosinophilia resolved 4 weeks after therapy, and the IgG reverted to negative after 3 months of completion of treatment. The patient continues to be monitored clinically for recurrence and is currently free of symptoms.

## 3. Case Discussion

The global burden caused by* Strongyloides stercoralis* is not known, with estimates of 370 million infected people worldwide [2]. The parasite is present in tropical, subtropical, and temperate areas [3]. Humans become infected when filariform (L3) larvae penetrate the skin or the oral mucosa. The larvae migrate through the blood stream to the lungs, and then they enter the airway, are swallowed, and eventually reach the small intestine where they mature into adult females capable of laying eggs without the aid of males (parthenogenetic females). The eggs are embryonated upon release and hatch internally. The resultant rhabditiform (L1) larvae are released in the stool to the external environment where they mature into filariform larvae. The rhabditiform larvae can also develop into free-living adult worms capable of producing infective filariform larvae. The crucial characteristic that sets* S. stercoralis* aside from other helminthic infections is the capability of the rhabditiform larvae to molt into infective filariform larvae in the intestine. These tissue-penetrating larvae can enter the circulation through the colonic wall or perianal skin and complete an internal cycle. This process, known as autoinfection, is the mechanism by which the infection is established for the life of the infected individual [4, 5].

Approximately 50% of patients with chronic infection have no symptoms. Nonspecific gastrointestinal complaints, pulmonary, and cutaneous symptoms are the most commonly reported manifestations in those that are symptomatic [4]. Hyperinfection usually develops in immunocompromised states when reduced immune surveillance leads to an unrestricted proliferation of worms through accelerated autoinfection. The distinction between autoinfection and hyperinfection is therefore quantitative rather than specifically defined. In our case, the respiratory complaints, abnormal imaging findings, and resolution of symptoms with treatment are highly suggestive of an accelerated autoinfection involving the lungs despite the failure to isolate larvae from a sputum sample. The nematode, larvae, and on occasions eggs can be detected in extra-intestinal regions like the lungs [6]. Pulmonary manifestations are the rule and patchy fleeting pulmonary infiltrates are usually seen. Diffuse bronchopneumonia and severe alveolar hemorrhage can lead to death [4, 6]. Disseminated strongyloidiasis is typically referred to the condition when worms are found in ectopic sites other than the lungs (e.g., brain). In hyperinfection and dissemination, enteric bacteria or fungi can be carried by larvae leading to potentially deadly metastatic infection elsewhere (e.g., pneumonia, meningitis) or septicemia [4, 6].

The incidence of* Strongyloides *hyperinfection after renal transplantation is unknown and believed to be uncommon. Hyperinfection typically occurs during the first 3 months after transplantation with mortality rates reaching up to 50% [6]. Although transmission of Strongyloides can occur from an infected renal allograft [7], most are the result of the uncontrolled proliferation of the nematode in an immunocompromised recipient with potential exposure before transplantation. In our case, the patient had eosinophilia for several years before transplantation making acquisition of the infection in the jungles of Vietnam likely.

Strongyloidiasis is known to be endemic in Vietnam [3]. For example, the prevalence of infection among Vietnam veterans ranges from 1.6 to 11.6% [8, 9]. For this reason and for the potentially deadly complications of untreated disease, refugees from South East Asia should receive preemptive treatment before arrival to the US. The Division of Global Migration and Quarantine of the Center for Disease Control and Prevention (since 2005) recommends prearrival ivermectin at a dose of 200 *μ*g/kg/day orally once a day for 2 days for those without contraindications [10]. Our patient arrived to the US before this recommendation was enacted.

The American Society of Transplantation recommends pretransplant screening with stool examination and* Strongyloides stercoralis* immunoglobulin G enzyme-linked immunosorbent assay (ELISA) antibody in recipients and donors from endemic areas or with eosinophilia [11, 12]. The tests have diagnostic limitations. Different serological assays have been evaluated with estimated sensitivities between 84% and 95% in chronically infected patients [13] with specificity being 100% with an acceptable cut-off for sensitivity [14]. Interpretation of positive results needs to be done with caution as cross-reactivity exists with* Ascaris lumbricoides* and* Schistosoma *spp. [5]. In addition, seropositivity can persist for years despite appropriate treatment and a negative result does not exclude the diagnosis. Molecular methods in diagnosing the infection are still limited owing to results from different studies showing these tests to be less sensitive than serological tests although it is still unclear if molecular tests are more sensitive compared to traditional fecal testing due to variability in different studies conducted so far [15]. Our patient was not screened or presumptively treated for this parasitic infection for unclear reasons.

Coprological examination alone has poor sensitivity (15–30% for a single specimen) due to the fact that larvae are excreted intermittently and in small quantities [5]. The sensitivity of a stool exam can increase by performing more laborious and expensive testing. For example, the sensitivity increases to almost 100% if 7 consecutive daily stool specimens are examined by experienced personnel [15]. Study by Sato et al. reported the agar culture plate method to have a sensitivity of 96% when multiple stool samples were tested, the detection rate being less than 60% if only single sample was tested [5, 16]. The goal of treatment is complete eradication of Strongyloides to prevent serious disease. Due to these limitations, presumptive treatment is recommended by some for patients with risk factors and negative serology and stool examination [6].

A definite test of cure does not exist since a negative stool agar culture plate does not guarantee cure and serology may remain positive for years despite a curative treatment course. It is important to remember that treatment failure or relapse is more common in immunocompromised patients and therefore treatment should be preferentially administered before transplantation [4, 6].

Ivermectin results in more people cured than albendazole and is at least as well tolerated. In trials of ivermectin with thiabendazole, both have similar cure rates but ivermectin is better tolerated [17]. The duration of therapy in immunocompromised patients is unclear. For chronic intestinal strongyloidiasis, ivermectin should be administered for a minimum of two weeks of daily therapy to extend over the full life cycle of the parasite, followed by posttreatment monitoring of stool samples and antibody titers to document clearance. Hyperinfection requires a longer course of treatment and current guidance dictates for ivermectin to be given until microscopic clearance of larvae from infected sites is documented [6].

## 4. Conclusion

Despite the high mortality rate and being a preventable and treatable entity, hyperinfection due to* Strongyloides* continues to be reported in renal transplant recipients. Comprehensive guidance regarding the prevention and treatment of parasitic diseases in the setting of solid organ transplantation has been published. It is imperative that healthcare professionals involved in the care of these individuals be cognizant of the importance to follow such recommendations.
